# Dynamic Biomechanical Analysis of Vocal Folds Using Pipette Aspiration Technique

**DOI:** 10.3390/s21092923

**Published:** 2021-04-21

**Authors:** Florian Scheible, Raphael Lamprecht, Marion Semmler, Alexander Sutor

**Affiliations:** 1 Institute of Measurement and Sensor Technology, UMIT-Private University for Health Sciences, Medical Informatics and Technology, 6060 Hall in Tirol, Austria; raphael.lamprecht@umit.at (R.L.); alexander.sutor@umit.at (A.S.); 2 Division of Phoniatrics and Pediatric Audiology, Department of Otorhinolaryngology-Head and Neck Surgery, University Hospital Erlangen Medical School, Friedrich-Alexander-University Erlangen-Nürnberg, 91054 Erlangen, Germany; Marion.Semmler@uk-erlangen.de

**Keywords:** pipette aspiration, vocal folds, laser vibrometry, elastic modulus, young’s modulus, frequency-dependent material properties, phonation, larynx

## Abstract

The voice producing process is a complex interplay between glottal pressure, vocal folds, their elasticity and tension. The material properties of vocal folds are still insufficiently studied, because the determination of material properties in soft tissues is often difficult and connected to extensive experimental setups. To shed light on this less researched area, in this work, a dynamic pipette aspiration technique is utilized to measure the elasticity in a frequency range of 100–1000 Hz. The complex elasticity could be assessed with the phase shift between exciting pressure and tissue movement. The dynamic pipette aspiration setup has been miniaturized with regard to a future in-vivo application. The techniques were applied on 3 different porcine larynges 4 h and 1 d postmortem, in order to investigate the deterioration of the tissue over time and analyze correlation in elasticity values between vocal fold pairs. It was found that vocal fold pairs do have different absolute elasticity values but similar trends. This leads to the assumption that those trends are more important for phonation than having same absolute values.

## 1. Introduction

Speech communication is the most common and probably the fastest way to communicate and transfer information in a high density between human beings. Almost 10% of the general population suffer from voice disorders in their lifetime [1]. It is assumed that phonotrauma is a result of high impact stresses between the vocal folds (VFs) [2]. Phonotrauma is known to be the reason for VF lesions, which can often cause hoarseness, more vocal effort or even the feeling of dyspnea while phonation [3]. To understand the function of VF, to detect lesions or even engineer VF-tissue, the mechanical properties are an important factor [4]. There are many published data of excised larynges but only little of in-vivo measurements [5,6,7,8]. As one can imagine, the collected data differ a lot between in-vivo and in-vitro, because the samples suffer from dehydration.

Therefore, the possibility of in-vivo measurements would open up possibilities for research and clinical applications. The pipette aspiration technique (PipAsT) does have the potential to be miniaturized and used in an endoscopic device for in-vivo measurements, similar to a device proposed by Tran et al., with the advantage of dynamic measurements [9]. Multiple studies on dynamic measurements on silicone samples can be found [10,11,12,13].

Using the modified pipette aspiration technique presented in this article, it is possible to combine classical static PipAsT with the ability of defining frequency-dependent properties. This is accomplished by compelling a harmonic movement, produced by a pressure signal and measuring the excitation of the sample.

The forced oscillation of VF was examined in previous studies, for example Chan et al. [14] show results of the elastic storage and loss moduli at a frequency of 75 Hz. In this and similar studies, anatomizing the tissue out of the larynx was necessary, which cost some effort and include the risk of changing the sample properties. Using the PipAsT, dissecting the VF is unnecessary, which reduces the time between death of the donor till examination drastically.

In the following paper, an improved version of the dynamic PipAsT was developed to measure on silicone and biological samples and results are presented.

## 2. Materials and Methods

In the following section, the material model and its properties, which are used in this work, will be explained. Further, the used silicon and biologic samples are described. Conclusively, we describe the pipette measurement setup, basically a device which is able to aspirate the underlying sample with a defined negative pressure and also to determinate the resulting displacement. Finally, the processing of the measured data is illustrated.

### 2.1. General Material Model

In this work, the material properties of specimens will be measured in different ways, but the underlying assumptions of the material model remain the same. The materials are described with the Hooke’s Law formulation

(1) $$\vec{\sigma }=A\vec{\epsilon },$$

with the stress $\vec{\sigma }$ equal to the strain $\vec{\epsilon }$ multiplied with the stiffness matrix ***A*** containing the elastic properties. Both silicone and vocal fold are assumed to be isotropic, incompressible, homogeneous and linear elastic. That results in a simplification of the matrix, so that the material can be described sufficiently with two parameters, the shear elastic modulus *G* and the Poisson’s ratio *ν* [15]. The Young’s modulus *E* is then given by

(2) E=2(1+ν)G.

### 2.2. Complex Elasticity Modulus

Applying a sinusoidal force on a surface, the stress curve is followed by a harmonic deformation which has the same frequency *f*, but may be out of phase [16]. In oscillation testing, not only the absolute deformation amplitude $d_{0}$ is measured, but also the phase shift Δϕ between deformation and the harmonic driving force. In this case, the pressure *p*, which equals the stress, and the displacement *d* of the tissue are described in time *t* with a sinusoidal function

(3) $$\begin{array}{c} p\left(t\right)=p_{0}\times sin\left(\omega t\right)=ℜ\;p_{0}^{*}e^{i\omega t} \end{array}$$

(4) $$\begin{array}{c} d\left(t\right)=d_{0}\times sin\left(\omega t+\Delta \phi \right)=ℜ\;d_{0}^{*}e^{i\omega t}, \end{array}$$

with the maximum amplitudes $d_{0}$ and $p_{0}$, the asterisk (*) identifies the variable as an element of the complex numbers, where only the real part *ℜ* is taken. The angular frequency is given by ω=2πf. The phase shift is present in the real and imaginary part of $d_{0}^{*}$. In classical rheology, the complex elasticity module is defined as

(5) $$E^{*}\left(i\omega \right)=\gamma \frac{d_{0}^{*}\left(\omega \right)}{p_{0}^{*}\left(\omega \right)}.$$

The constant $\gamma =Cd_{i}$ was added to include pipette parameters, which will be described in Section 2.3.2. The complex expression can be divided in two parts

(6) $$E^{*}\left(i\omega \right)=E^{\prime }\left(\omega \right)+iE^{\prime \prime }\left(\omega \right),$$

the storage modulus $E^{\prime }$ and loss modulus $E^{\prime \prime }$. Calculated with the absolute value $E_{0}$ and the trigonometric functions those moduli can be written as,

(7) $$\begin{array}{ccc} E^{\prime }=E_{0}\;cos\left(\Delta \phi \right) & & \mathrm{storage}\mathrm{modulus} \end{array}$$

(8) $$\begin{array}{ccc} E^{\prime \prime }=E_{0}\;sin\left(\Delta \phi \right) & & \mathrm{loss}\mathrm{modulus}, \end{array}$$

where the phase shift defines the ratio between both moduli [16]. In case of PipAsT, the absolute elastic modulus is calculated with Equation (11), for a correct notation the factor γ is present in Equation (5).

### 2.3. Measurement Principles

#### 2.3.1. Static Measurement

The static Young’s modulus is measured by indentation measurements, where the pipette is used as indenter. The indentation stiffness is given by the Bulychev–Alekhin–Shorshorov (BASh) relation and can be written as

(9) $$F=2M_{3}a\delta$$

where δ is the displacement of the flat-ended indentation (radius *a*) under the contact force *F* [15]. For simplification reasons, the pipette is regarded as a simple flat-ended indenter without an inside hole. $M_{3}$ represents an elastic constant, that includes the Young’s modulus *E* and the Poisson’s ratio ν by

(10) $$M_{3}=\frac{E}{1-\nu^{2}}.$$

#### 2.3.2. Pipette Aspiration Technique

**Figure 1:**
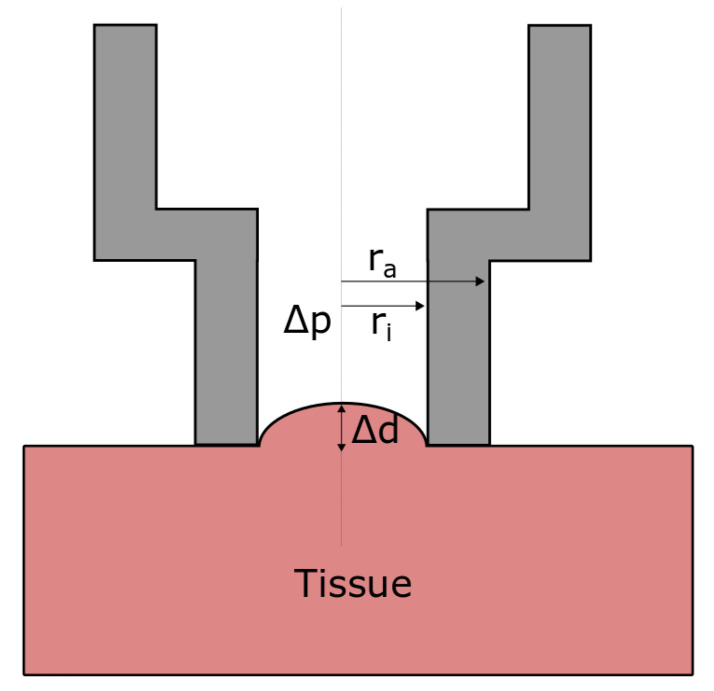
The principle of pipette aspiration technique. The negative pressure difference Δp aspirates the tissue in the center of the pipette to the displacement Δd.

First application of PipAsT on soft tissue was done by Aoki et al. [17]. The pipette is placed on the specimen which is sucked into the pipette by a negative pressure, as can be seen in Figure 1. Using static measurements, it is common to calculate the Young’s modulus $E_{0}$ by using the Aoki-formula, where variations in pressure Δp and displacement Δd are set into ratio. Further, the pipette diameter $d_{i}$ and a coefficient *C*, which depends on its wall thickness, are considered. Based on the assumptions that the specimen is isotropic, incompressible, homogeneous and linear elastic, the Aoki-formula is used to determine the Young’s modulus [17]

(11) $$E_{0}=C\frac{\Delta p}{\frac{\Delta d}{d_{i}}}.$$

The therefrom obtained Young’s modulus represents an average of the sample under the pipette within a depth of one pipette diameter $d_{i}$ [17]. In case the examined sample is inhomogeneous in this area, the thicker layer is the dominate one and the measured value represents the elasticity of that part of the sample [18]. In this study, the dynamic elastic moduli are calculated with this formula.

In order to calculate the complex Young’s modulus, the absolute value $E_{0}$ is multiplied with the corresponding trigonometric function with the corresponding phase shift. The phase shift Δϕ is defined by cross-correlating the pressure signal and the velocity signal. The peak of the resulting data indicates the lag between both signals in samples Δn, knowing the sample-rate and the frequency, the phase shift in the time domain is calculated. The used hardware allows a sample-rate of 51.2 kHz which results in a resolution of ±Δϕ=0.12π for the highest frequency of 1000 Hz. Due to the fact that the velocity signal is measured but the displacement is of interest, a π/2 shift is added, which is necessary due to the integration of the sinusoidal shaped signal [19]. In Section 2.5.2, the measurement devices and methods are described in detail.

### 2.4. Samples

To prove the concept and defining measurement accuracy, it is important to have a stable sample, therefore measurements are taken on silicone samples initially. Porcine VFs were chosen as biological samples.

#### 2.4.1. Silicone Samples

The squared silicone samples with a dimension of 50 × 50 × 15 mm made of silicone (Ecoflex 0030, Smooth-On Inc., Macungie, PA, USA) and variable volume proportions of thinner (from the same company), have elasticity values in the range of porcine vocal folds, which vary from 1.6–5.7 kPa referring to Amir et al. [2] and 16.3 kPa ± 2.9 kPa measured by Alipour et al. [6]. The static elasticity values of the samples have been measured with the flat-ended indentation study, results can be seen in Table 1. The Poisson’s ratio of the silicone samples was defined by Ilg et al. [11] as ν=0.499. This value was used to calculate the static Young’s modulus. With a Poisson ratio close to 0.5, the silicone can be assumed as an incompressible material, and since we measure in the linear elastic range of the silicone, all assumptions for the Aoki formula (Equation (11)) are satisfied.

#### 2.4.2. Vocal Fold Tissue Samples

**Figure 2:**
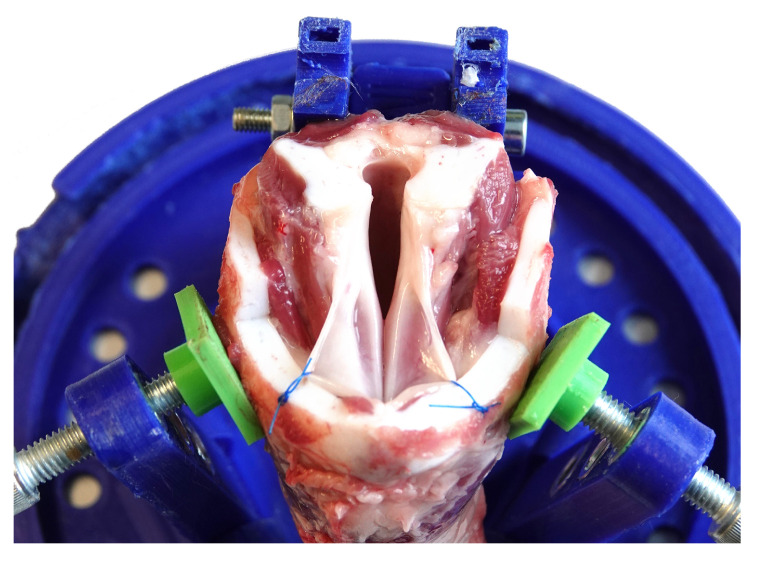
Prepared larynx in holder ready for the measurements. The larynx is held in place by two screws which are fixating the cricoid cartilage against the back plane. To expose the vocal folds and make them accessible more easily, the vestibular folds are sewn to either side.

Porcine VFs were used for measurements which were provided by a local slaughterhouse (Mayer, Natters, Austria). The animals have been killed for food purpose and had roughly the same age. A potential risk of tissue degeneration was avoided by keeping the time till measurements as short as possible. They were picked up shortly after death and were prepared for measurements. Thanks to the cooperation and the short transportation chain, it was not necessary to quick freeze the larynges. Therefore, measurements on larynges approximately 4 h postmortem were possible. Four different porcine larynges have been prepared for measurement. To make measurements in the center of the anterior-posterior line of the VFs possible, otiose tissue around the VF was removed and the vestibular folds were pulled to the side by some stitches, as can be seen in Figure 2. One of the four samples could not be used for the measurements because the VFs were damaged during dissection. To protect the samples against dehydration between measurements, the samples are covered by a cloth soaked with NaCl-solution and put in to refrigeration at 4 °C overnight.

### 2.5. Measurement Setup

The developed measurement setup is capable of determining the static and dynamic elasticity values of soft tissue. In the following, the two procedures and the used hard- and software are explicated.

#### 2.5.1. Static Elasticity Measurement

**Figure 3:**
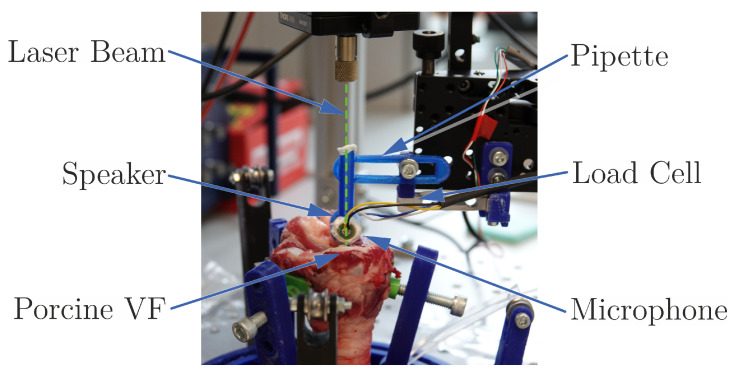
The experimental setup during measurements on a porcine larynx. The pipette is mounted on a load cell, which estimates the contact force. A speaker on the hidden side is producing the pressure signal, which is measured by the built-in microphone. The laser-beam (dashed green line) is projected through the pipette and reflected on the VF surface.

The pipette unit, consisting of the parts shown in Figure 3, is mounted to a gripper arm, which enables a positioning with many degrees of freedom. The distance between unit and gripper arm is measured with a linear potentiometer (KTC 100P, IXTHUS U.K. Sensors, Towcester, UK) and can be changed by an adjusting screw. The load cell (TAL221, HT-Sensors, Xi’an, China) measures the acting force *F*. The distance is increased, which forces the tissue to displace, conclusively the static elasticity can be calculated through the measured values by using Equation (2).

#### 2.5.2. Dynamic Pipette Aspiration Technique

**Figure 4:**
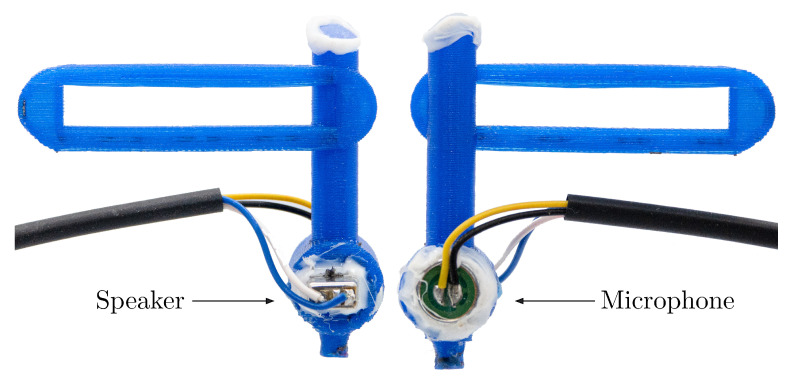
Detailed illustration of the pipette, where both sides are shown. Speaker and microphone are mounted face to face.

Based on the work of Weiss et al. [20] and Maghzinajafabadi et al. [13], the dynamic elasticity measurements of soft tissue using pipette aspiration have been further developed. Figure 3 and Figure 4 show the measurement setup with the 3D printed pipette, which is pressed at the middle of the anterio-posterio line of the VF, with a certain force, estimated by the mentioned load cell. A speaker (CI-22955-000, Knowles, Itasca, IL, USA) produced sinusoidal pressure exciting the tissue. Its velocity is determined by a laser beam that permeates through the pipette. The laser beam is produced by a single-point vibrometer (OFV-5000 & MLV-I-120 & MLV-O-SRI, Polytec, Waldbronn, Germany) which calculates the velocity of the reflecting tissue based on the Dopplershift of the interfering laser beams. It is used with a fiberglass lens (OFV-130-x, Polytec, Waldbronn, Germany) which allows smaller distances to the sample and easier positioning.

Conclusively, the absolute deformation is calculated by integration. A calibrated microphone (CMC-9745-103T, Cui Devices, Lake Oswego, OR, USA) senses the exact pressure in the pipette. Due to the small volume in relation to the wavelength of the produced sound, it can be assumed that the measured pressure is equal to the pressure exciting the tissue.

**Figure 5:**
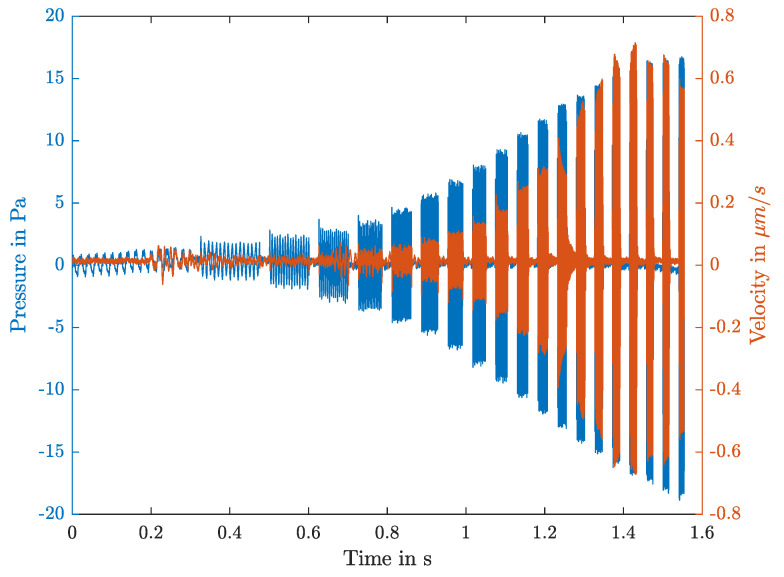
The applied pressure signal (blue) and resulting surface velocity (orange) plotted over time. The pressure signal excites the tissue at each frequency from 50–1000 Hz for 10 periods. The speaker driving voltage is kept constant, due to the speaker’s frequency-dependent sensitivity the pressure level is increasing.

To excite the tissue a pressure signal with ten periods of each frequency varied between 50 Hz to 1 kHz was produced with a step width of 50 Hz. In Figure 5, pressure and velocity are shown.

In order to excite the tissue with different pressure levels, this process is repeated for three different driving voltages of the speaker. Due to the speaker’s frequency-dependent sensitivity, the pressure amplitudes increase with the frequency up to 9 Pa, 14 Pa and 19 Pa, respectively. The pressure amplitudes values are 113, 117 and respectively 120 dB SPI. See Figure 5 where the data for the biggest excitation amplitude are shown. The pressure and velocity peak height of each frequency are determined through a Fast Fourier Transformation (FFT) over each frequency interval. The total length of the pipette is 50 mm, the produced pressure acts through the 1.25 mm opening on the bottom side and the outer radius of the pipette is 2 mm. This results in a wall thickness coefficient of C=1 [17].

### 2.6. Measurement Procedure and Data Evaluation

**Figure 6:**
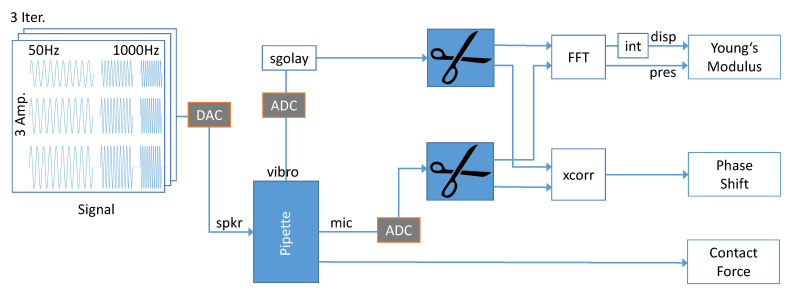
Algorithm architecture: The speaker (spkr) is driven at a harmonic signal with 10 periods per frequency, for 3 different amplitudes the signal is repeated in 3 iterations. The microphone (mic) measures the produced pressure difference and the vibrometer (vibro) registers the velocity of the tissue. After digitizing and filtering the signals, the signals are cut into sequences corresponding to the frequency. In that way, the Young’s modulus is calculated by determining the maximum displacement (disp) and pressure values (pres) by Fast Fourier Transforming these sequences. Additionally, the phase shift is evaluated by cross correlating those signals. The contact force is specified for each measurement with the load cell.

Signal production and data acquisition are managed on the software side with Matlab (2019b, MathWorks, Natick, MA, USA) and on the hardware side with National Instrument Cards (NI92623 and NI9215, National Instruments, Austin, TX, USA) with a sample-rate of SR = 51,200 Hz. The measurement procedure is schematically drawn in Figure 6.

Every measurement includes three iterations, in this way it is possible to identify the quality of measurement by determining the standard deviation and potential outliers can be removed.

The speaker produces the pressure signal in the pipette, where the microphone and the vibrometer detect the obtained pressure and the resulting movement; after converting the analog signals, the data are evaluated with Matlab.

First, a Savitzky–Golay filter, with an order of 9 and a window length of 0.957 ms, is applied to the velocity signal, to reduce noise.

As a result that the steady-state is of interest, the first period of the signals, which are needed to settle, are cut for each frequency. In order to do so the Matlab function *findpeaks()* is used to locate pressure peaks, and the signal is cut before and after the first and last two peaks of each frequency. The maximum values of each frequency-segment are received through a Fast Fourier Transformation (FFT), realized by the Matlab function *fft()*. After integrating the maximum velocity difference, Δv is calculated analytically by $\Delta L=\frac{\Delta v}{2\pi f}$, to get the total displacement ΔL the Young’s modulus, which is calculated in Equation (11). The cross correlation between the velocity and pressure signal delivers the phase shift between excitation and resulting movement. By producing 10 periods, a spectral resolution of Δf=1/10×SR= $1.95\times 10^{-6}$ Hz can be achieved [21].

Studying the deviation between measurements of different amplitudes, it can be checked if the applied pressure range forces the tissue to move outside its linear behavior. By checking the deviation between different iterations, the quality of the measurement can be evaluated. Some outliers between iterations are deleted with the Matlab function *rmoutliers()*.

## 3. Results

The following sections present the results of the silicone and the VFs separately. On silicone samples, concept finding investigations like the effect of the contact force as well as studies of the reproducibility and measurement precision were carried out.

### 3.1. Silicone

#### Effect of the Contact Pressure on Pipette Measurement

The contact pressure between pipette and specimen does affect the measurement. In order to observe this effect, the pipette is pressed on the sample, the displacement and force are logged and the dynamic elasticity is measured for each force step.

**Figure 7:**
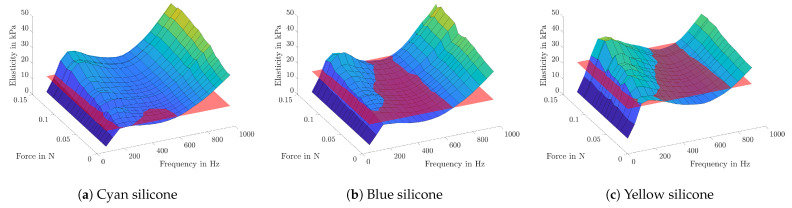
For different pipette contact pressures, the elasticity values are plotted against the applied frequency. The red plane symbolizes the static elasticity of each sample, which are 6.04 kPa for cyan (**a**), 11.56 kPa for blue (**b**) and 18.77 kPa for yellow silicone (**c**).

In Figure 7, the measurement results are shown. Elasticity values are plotted in the Z-direction against the corresponding frequency on the X-axis and the applied contact force on the Y-axis. Additionally, the transparent red plane symbolizes the static Young’s modulus of each sample. It can clearly be seen that higher contact forces lead to higher elasticity values, due to the increased pretension.

Further, the examinations show that all samples do have a local minimum between 400 and 600 Hz, followed by a steep rise for higher frequencies. When increasing the contact force, this frequency-dependent trend stays unchanged, but with an almost linear offset rise.

### 3.2. Reproducibility

In order to investigate the reproducibility of the PipAsT, a series of separate 14 measurements on silicone samples was made. Therefore, the position on and the inclination to the sample was kept the same, but the pipette was placed each time anew with a similar contact force of 0.1017 N ± 0.0045 N.

**Figure 8:**
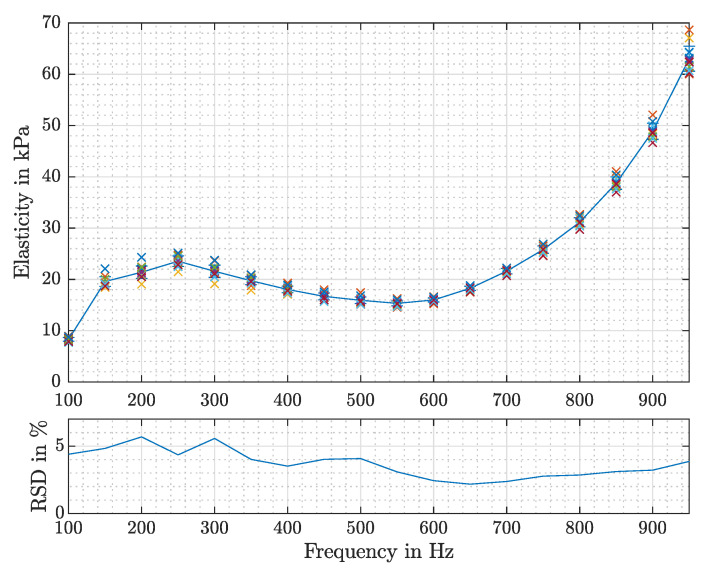
The measured elasticity values for each frequency are marked with crosses. The mean value is plotted with the solid line. Beneath, the relative standard deviation (RSD) is shown in percent.

The results are shown in Figure 8, where the frequency-dependent elasticity is illustrated for each measurement. Further the relative standard deviation (RSD) is presented, which reaches a maximum value of 5.68% at 200 Hz.

### 3.3. Measurement Precision

**Figure 9:**
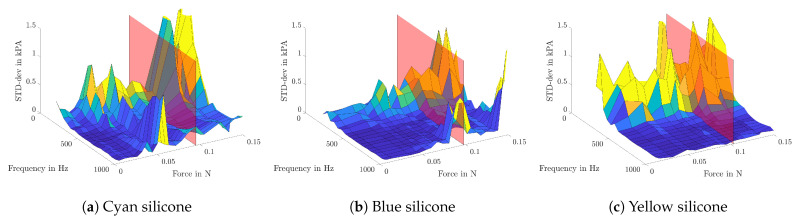
For different pipette contact pressures, the standard deviations (STD-dev) of the elasticity measurements are plotted against the applied frequency. The red plane tags the contact force of about 0.1 N, which was chosen. The results for the cyan, blue and yellow silcone are shown in subfiguire (**a**–**c**).

Due to the fact that there is no possibility to verify the measured values with others, only the precision of the measurement, but not the accuracy, can be determined. Therefore, the standard deviation of the Young’s modulus between multiple iterations and excitation pressures is calculated. In Figure 9, the results for all frequencies and contact forces are shown. The red plane symbolizes the contact force of 0.1 N, which was chosen for further examinations. The exact values can be taken from Table 2.

It can be seen that there is a higher standard deviation (STD-dev) for lower frequencies, which results from the poor speaker performance in this frequency range, see Figure 5. Apart from that, lower STD-dev values at a contact force of about 0.1 N are noticed.

On the one hand, low contact forces can result in fluttering tissue, where the excited movement propagates out of the pipette and the assumptions, used in order of Equation (11), do not hold anymore. On the other hand, high contact force leads to a pretension of the tissue and could even harm it if the resulting displacement is far beyond the linear elastic range. It can also be seen that the standard deviation between measurements is getting bigger for greater contact forces, as can be seen in Figure 9.

**Figure 10:**
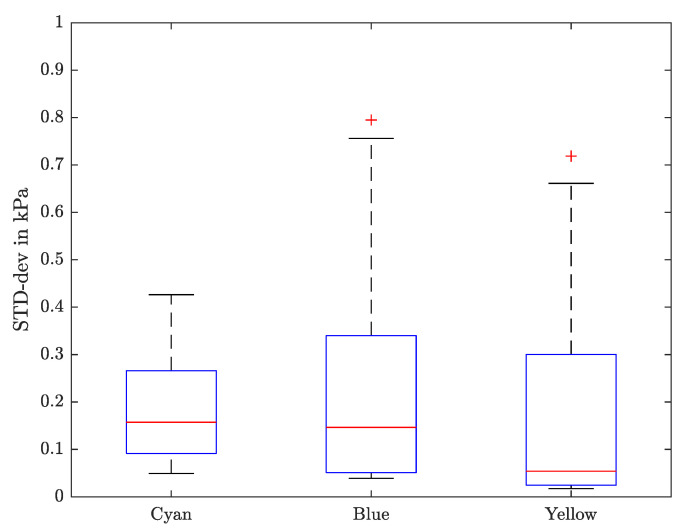
The standard deviation (STD-dev) for each sample with a contact force of about 0.1 N is shown in a box plot, in which the median value is indicated as red line.

A contact force of around 0.1 N shows a solid trade-off and is aimed for further measurements. For an easy comparison, the STD-dev for each sample is shown in the boxplot in Figure 10.

The measurements on silicone show a maximum STD-dev of 0.795 kPa for low frequencies and on average 0.198 kPa over all frequencies.

#### Phase Shift and Complex Elasticity

**Figure 11:**
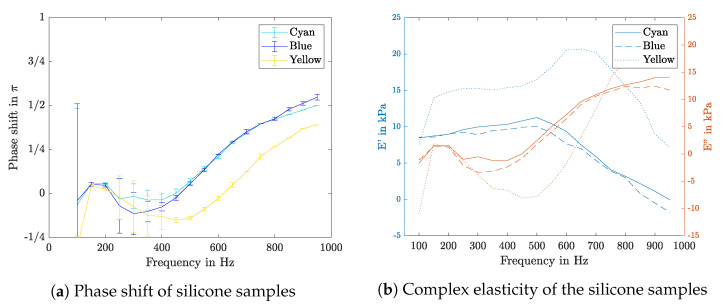
The phase shift (**a**) and complex elasticity (**b**) values over the frequency range of the different silicone samples.

By cross correlating the pressure signal with the sample movement, the phase shift is determined. The results of the silicone samples are shown in Figure 11a, in which a negative phase shift between 200 and 600 Hz can be seen. Further, the phase shift rises up to quarter of a period for high frequencies. The resulting complex elasticities are shown in Figure 11b.

### 3.4. Vocal Fold Tissue

After concept proofing measurements on silicon samples, the PipAsT is applied to porcine VF tissue, which are considered from this point on.

#### 3.4.1. Comparison of Frequency-Dependent Elasticity Trends between Different Larynges

**Figure 12:**
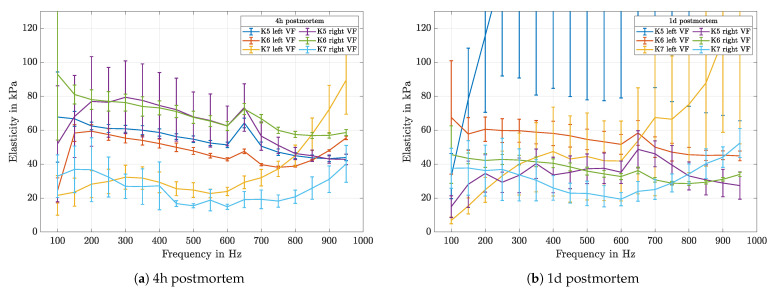
The elasticity values are plotted for each frequency. The measurements were taken 4 h and 1 d postmortem and are shown in subfigure (**a**) respectively (**b**). For the left VF on sample K5 the measured values rise up to 180 kPa, for an intuitive comparison the axis is kept the same.

The measurements were taken in the center of the anterior-posterior line of the VFs with a mean value of all contact forces of 0.1 N. Figure 12a shows the mean of multiple measurements with different driving amplitudes for each frequency. As the tissue shows linear behavior in the observed pressure range, this is well justified.

It can be seen that elasticity values vary in the range of 15–92 kPa when measured four hours after death. This agrees with the results reported from earlier investigations [6,22]. The measurements were repeated one day later. The results, see Figure 12b, show a changed elasticity in the range of 10–180 kPa. Not only a change in elasticity can be seen but also a loss in accuracy, indicated by bigger errorbars. Only sample *K5* shows outstandingly high elasticity values, which do not fit to the other samples nor to the opposite VF, which leads to the presumption of strong dehydration during storage.

Elasticity values of left and right VF pairs differ a lot and do not show similar values. Two of three samples show a significant peak at a frequency of 650 Hz. In former measurements with the silicone samples, this effect did not occur. It was also not possible to reproduce this peak in later measurements, therefore it has to be a peculiarity of those samples.

**Figure 13:**
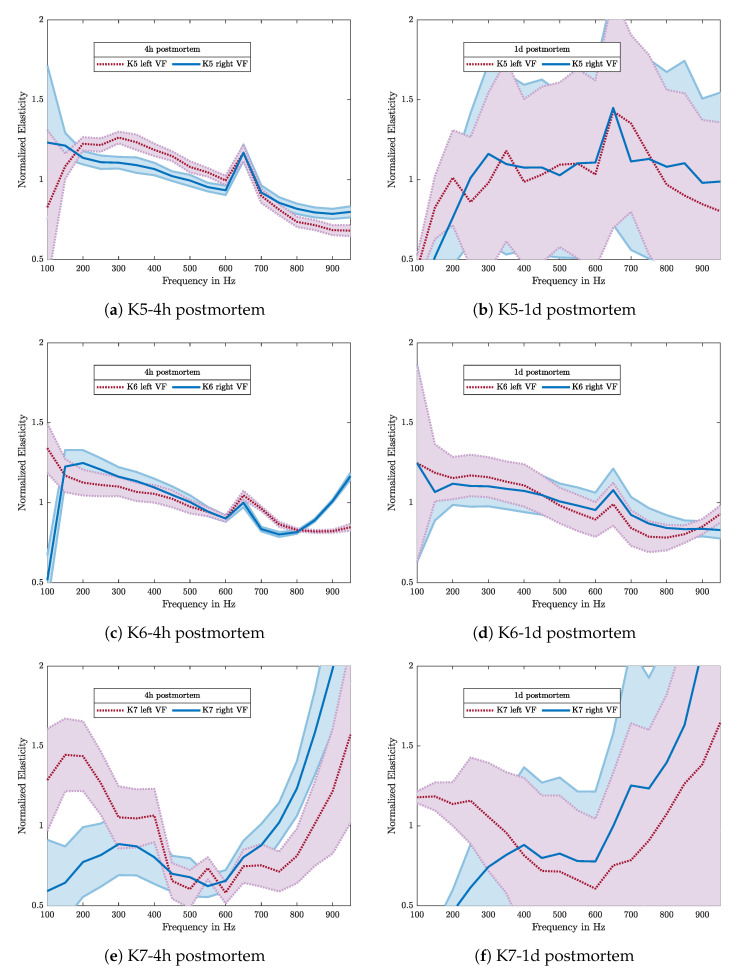
The normalized elasticities of samples K5, K6, K7 and their standard deviation (shadowed band) are shown, in which the left column indicates the results 4 h and on the right one 1 d postmortem. Same elasticity characteristics over frequency can be found within same VF but at different instants of measurement time.

Regarding the development of the elasticity due to the aging of the tissue, some significant changes can be seen. Comparing the data in Figure 12a and the ones measured one day later in Figure 12b, example K7 right side and K6 left show quite the same absolute values. K6 right shows similar frequency-dependent characteristics; even their offset changed significantly. A general claim about a trend in the temporal development of the tissue cannot be made. It also can be seen that values between left and right VF differ a lot, and related pairs do not show similar values. To observe the frequency-dependency in general, the elasticity values have been normalized with the corresponding mean over the frequency range. As can be seen in Figure 13, same VF-pairs show the same characteristics also 1 d postmortem.

#### 3.4.2. Comparison between VF Pairs

**Figure 14:**
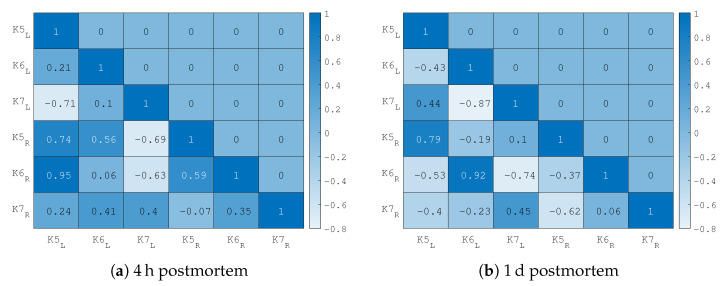
Correlation values between frequency characteristics of different VFs (normalized curves shown in Figure 13). The results for the measurement 4 h are shown in the subfigure (**a**) and 1 d postmortem in subfigure (**b**). The saturation of the cell indicates the value of the correlation coefficient.

The normalized elasticity values of all measurements were evaluated with the correlation Matlab function *corrcoef()* and the results can be seen in the heat map of Figure 14. To evaluate the similarities in frequency-dependent behavior, the Pearson correlation coefficient ρ is calculated with the Matlab function *corrcoef* (The Pearson correlation is defined as $\rho \left(A,B\right)=\frac{1}{N-1}\sum_{i=1}^{N}\left(\frac{A_{i}-\mu_{A}}{\sigma_{A}}\right)\left(\frac{B_{i}-\mu_{B}}{\sigma_{B}}\right)$ where the measurement *A* and *B* with a length of *N* samples with their standard deviations $\sigma_{A/B}$ are taken in account [23]). For measurements 4 h after death, the data between pairs do not show a strong correlation, especially sample K6 does not match. The correlation one day after death the correlation is very clear.

#### 3.4.3. Aging of Tissue

**Figure 15:**
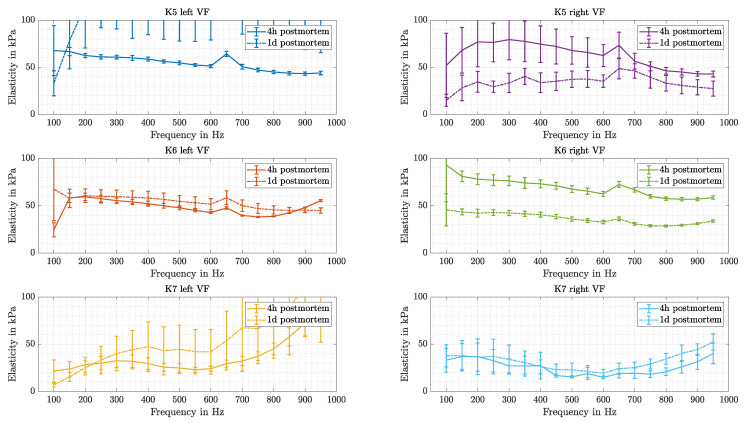
Elasticity changes over time for each sample (K5, K6, K7) and both VF sides are compared (left and right column). Measurements are taken 4 h and 1 d postmortem.

For each side of all VF samples, the change in elasticity values between the measurement 4 h and 1 d postmortem are compared. This can be seen in Figure 15.

#### 3.4.4. Phase Shift

**Figure 16:**
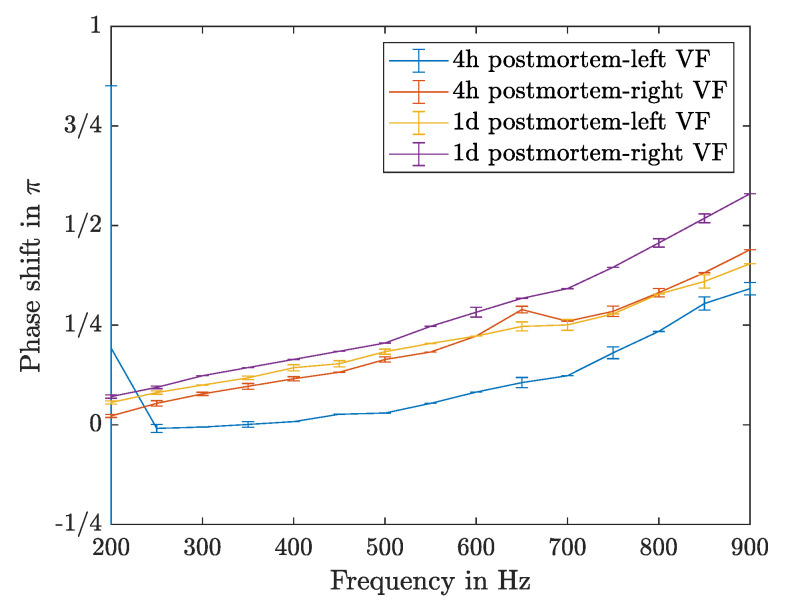
Phase shift of different measurements on sample K6.

The increase of driving frequency leads to an increase of the phase shift following almost a linear relation; this can be seen in Figure 16. The measurement of the left VF four hours postmortem shows the same linear trend with in general lower values.

#### 3.4.5. Complex Elasticity

**Figure 17:**
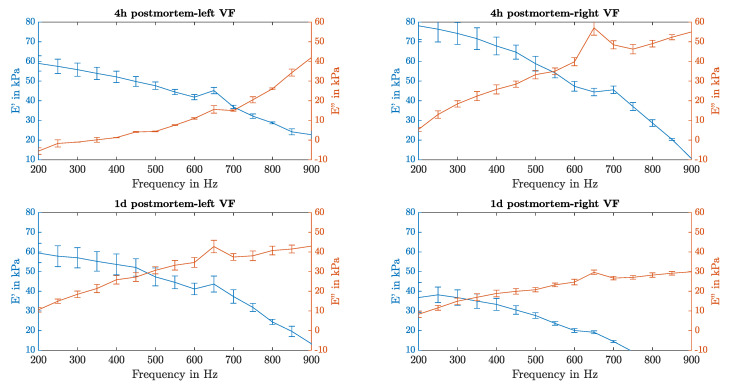
Complex elasticity of the sample K6: Measurements 4 h and 1 d postmortem (upper and lower row) for each VF side are plotted separately (left and right column). The storage modulus $E^{\prime }$ is given on the left Y-axis (blue) and the loss modulus $E^{\prime \prime }$ on the right (red).

To demonstrate the calculation of the complex elasticity, sample K6 is chosen as an example. Figure 17 is showing the complex elasticity values for each frequency for the VF pair 4 h and 1 d postmortem.

Regarding the evolution over time, on both VFs a flattening of the loss modulus $E^{\prime \prime }$ in higher frequencies can be seen in the data taken 1 d postmortem. Furthermore, it can be noticed that the frequency where the storage and the loss modulus crosses is not the same between VF pairs and this crossing shifts into lower frequencies for measurements taken 1 d postmortem.

## 4. Discussion

The following discussion is divided in three parts: first, the measurement technique in general will be reconsidered, then the used materials are discussed and lastly the results are reviewed, compared and verified with existing results.

### 4.1. Measurement Technique

For a reliable measurement, an enclosed space between pipette and tissue has to be established, hence the effect of contact pressure was studied on silicone samples, which show similar static elasticity values compared to the VF tissue. On the one hand, fluttering or other detaching leads to unusable results, on the other hand, a firm contact may lead to pretension of the tissue (Figure 7) or even damaging of the specimen. Contact forces of 0.1 N seem to be a good trade-off between both sides and the study of standard deviations shows on all samples slightly lower elasticity values for this force.

The measurement quality is depending on the vibrometer signal quality and therefore on the reflectivity of the sample tissue. The quality differs a lot between samples and also on its humidity.

This can be seen on the measurement data in terms of bigger standard deviation values, see Figure 12b. It has been observed that reflectivity correlates with the aging of the tissue, when the measurements carried out 4 h and 1 d postmortem are compared. Further investigations will be done to study the aging of tissue and its effect on dehydration and reflectivity.

Regarding the hardware selection, following insights are gained. Under 150 Hz, the used speaker does not deliver a clear signal which results in strongly varying data. This can be seen in most of the measurements. Therefore, it was not possible to detect the phase shift on the lower frequencies and the complex elasticity is shown only above 200 Hz. Regarding the speakers small formfactor, this is a disadvantage that can not be alternated. The vibrometer does have a fiberoptic used with the MLV-O-SRI lens, that allows near field measurements in various and easily changeable positions. Looking towards a miniaturization, for in-vivo measurements, a special lens with even shorter focal length would be necessary. The microphone delivers, after calibration, good measurements in the applied pressure range. The used load cell works sufficiently in this setup and the achieved results are valid for the application, but for miniaturization it is not small enough.

### 4.2. Material

The Aoki formula (Equation (11)) is based on the assumptions (linear, isotropic, homogeneous and incompressible), which are fulfilled by the silicone samples in the applied pressure range. In the following, it will be discussed whether the assumptions still hold for VF tissue, whether it is justified to use this formula and whether the calculated values can be trusted.

The measurements show that within the applied pressure range (1.5–19 Pa) the VF tissue does have a linear behavior.

The VF tissue is surely non-isotropic, but its upper layers do have an orthotropic structure [24]. Hence, it is possible to achieve reproducible results using the same surface inclination. Therefore, it has to be made sure that the pipette is placed orthogonal to the tissue’s surface.

The more concerning point is the inhomogeneous structure of the VF. The measured Young’s modulus represents an average of the vulume fraction underneath the pipette with a depth of one pipette diameter. Multi-layer structures are present, the thicker layer dominates the result [17,18]. In case of VF, the epithelium and the lamina propria, which are the connected outer layer and the first layer underneath, are affected [2,25]. As the epithelium’s thickness is negligibly thin, the measured value can be referred to the lamina propria. To be sure which layers of the VF are affected and measured, the depth of the laser penetration has not been determined nor taken into account. In the near future, investigations are planned to get a closer understanding of this effect.

A Poisson’s ratio of 0.5 has been assumed, according to literature, therefore the material is seen as incompressible [26].

### 4.3. Results

By comparing VF-pair’s elasticity, they showed different absolute elasticity values, but a similar frequency-dependent characteristic where a correlation could be shown.

This leads to the assumption that for phonation, the frequency-dependent characteristic of VF-pairs is more important than the same absolute elasticity values.

Results issued by Cochereau et al. [27] also show different elasticity between VF pairs in tension measurements and thus substantiate our results. We want to point out that this conclusion is based on the measurement of three larynges and does not have the statistical power to make general claims. A bigger series of tests is planned in the near future to give a better picture of that.

No significant trend in the aging of the tissue can be seen in our data. Dehydration affects the elasticity of biological tissue strongly, e.g., canine VF increased Young’s modulus up 4 to 7 times compared to in situ measurements [28]. Our suspicion would be that the effect of dehydration is much stronger than the effect of aging, thus further investigations with more measurements in time and maybe additional measurements to observe the dehydration are necessary to make a statement.

Additionally, measuring the phase shift opened a whole new spectrum of measured material properties. These results give information about loss and storage modulus and further estimations about the damping and the location of the eigenfrequency could be made.

## 5. Conclusions

Dynamic pipette aspiration technique was further developed with an algorithm that allows a fast measurement (1.5 s) over frequencies from 100–1000 Hz and multiple amplitudes and iterations. Further, it was checked if the measurement was carried out in the linear range of the sample and the goodness of the measurement is indicated. In that way, the elasticity as well as the phase shift between excitation pressure and responding tissue movement can be studied. The effect of different contact forces on dynamic elasticity values was studied on silicone samples, hence a contact force of 0.1 N could be shown to be a good trade-off for tissues with similar static elasticity value like VF. Measurements showed a maximum standard deviation of 0.795 kPa for low frequencies, in average over all frequencies 0.198 kPa.

Dynamic PipAsT was applied to multiple porcine vocal folds in order to analyze their elasticity behavior in a frequency range of 100–1000 Hz. The measurement was carried out on both vocal fold sides 4 h and 1 d postmortem to investigate the relation between both sides and the effect of tissue aging. A correlation in elasticity-trend between vocal fold pairs could be found, even the sides show different absolute values. This leads to the conclusion that for phonation the frequency-dependent characteristic of VF-pairs are more important than having the same absolute values.

It has been shown that a dynamic pipette aspiration technique was developed further towards a miniaturized device for clinical application and does deliver reliable results on multiple tissue samples.

## Figures and Tables

**Table 1:** Measured Young’s modulus by indenter compression on silicone pads.

	*E* (kPa)	*G* (kPa)
Cyan	6.04±0.80	2.03±0.26
Blue	11.56±1.10	3.88±0.37
Yellow	18.77±0.38	6.30±0.13

**Table 2:** Applied contact forces for each silicone sample.

Sample	Contact Force in N
Cyan	0.095
Blue	0.098
Yellow	0.103

## Data Availability

Not applicable.

## Abbreviations

The following abbreviations are used in this manuscript:

PipAsT	Pipette Aspiration Technique
NaCl	Sodium Chloride
STD-dev	Standard Deviation
RSD	Relative Standard Deviation
VF	Vocal Fold
DFG	German Research Foundation
FWF	Austrian Science Fund
